# Immune Responses and Protective Efficacy of Nanoemulsion-Adjuvanted Monkeypox Virus Recombinant Vaccines Against Lethal Challenge in Mice

**DOI:** 10.3390/pathogens14121293

**Published:** 2025-12-16

**Authors:** Congcong Zhang, Nuo Liu, Yanqi Zhao, Zhendong Pan, Dawei Wang, Wanda Tang, Yanhua He, Xu Zheng, Zhongtian Qi, Xinxin Zhang, Ping Zhao

**Affiliations:** 1 Department of Microbiology, Faculty of Naval Medicine, Naval Medical University, Shanghai 200433, China; luxion0227@gmail.com (C.Z.); liunuo3527@163.com (N.L.); panzhendong@smmu.edu.cn (Z.P.); grabbydowa@sina.com (D.W.); wandat22@163.com (W.T.); yanhua0556@163.com (Y.H.); zhengxu87@nankai.edu.cn (X.Z.); qizt@smmu.edu.cn (Z.Q.); 2 Key Laboratory of Biosafety Defense (Naval Medical University), Ministry of Education of the People’s Republic of China, Shanghai 200433, China; 3 Shanghai Institute of Materia Medica, Chinese Academy of Sciences, Shanghai 201203, China; yqzhao@baridd.ac.cn

**Keywords:** monkeypox virus, vaccinia virus, recombinant vaccine, nanoemulsion adjuvant, neutralizing antibody, lethal challenge, histopathology

## Abstract

The ongoing global monkeypox outbreak since 2022 has highlighted the urgent need for vaccine development. Current vaccination strategies rely on cross-protective immunity provided by orthopoxvirus-based live-attenuated vaccines. However, these vaccines not only exhibit suboptimal efficacy against monkeypox virus (MPXV) but also raise safety concerns, particularly given the significant global overlap between MPXV infections and HIV. Owing to their superior safety profile and accessibility, recombinant subunit vaccines represent a highly promising platform for monkeypox vaccine development. In this study, we developed a subunit vaccine comprising A29L, B6R, and M1R antigens formulated with a proprietary nanoemulsion adjuvant and evaluated its immunogenicity and protective efficacy. In mice immunized with a prime-boost regimen of the three individual antigens combined with the nanoemulsion adjuvant, comparable serum IgG levels against each antigen were elicited. Both A29 and M1 formulations induced serum antibodies with potent neutralizing activity against MPXV and Vaccinia virus Western Reserve strain (VACV-WR). Notably, M1 antiserum exhibited stronger neutralization than A29 antiserum, whereas B6R immune serum showed no significant neutralizing activity. Splenocytes from B6R-immunized mice mounted a robust IFN-γ response, which was markedly lower in those immunized with A29 or M1. All three monovalent vaccines conferred complete survival following an intranasal lethal MPXV challenge, with M1 providing the strongest protection. In a lethal VACV-WR challenge model, only M1 immunization conferred significant protection. Histopathological analysis of lung tissues on day 5 post-infection revealed more pronounced inflammatory features in B6R-immunized mice compared to the nanoemulsion adjuvant control group. Furthermore, the nanoemulsion-adjuvanted bivalent A29L + B6R formulation induced significantly higher IgG and neutralizing antibody titers and demonstrated superior protective efficacy compared to the aluminum hydroxide-adjuvanted formulation. This comparative preclinical evaluation provides important evidence to support the development of a safe and effective subunit vaccine against monkeypox.

## 1. Introduction

Monkeypox virus (MPXV), an enveloped double-stranded DNA virus within the *Orthropoxvirus* genus, causes monkeypox, a zoonotic disease [1,2]. Previously confined to regions of Central and West Africa, MPXV gained global attention during the 2022 outbreak, which revealed a shift in transmission dynamics through sustained human-to-human spread in numerous non-endemic countries, elevating it to a public health priority [3,4,5]. Since mid-2022, mpox has expanded globally, with more than 100,000 confirmed cases reported from over 120 countries by mid-2024 and over 150,000 cases from 127 countries by mid-2025, highlighting the urgent need for effective vaccine development [6,7,8]. Current vaccination strategies against MPXV rely on cross-protective immunity conferred by orthopoxvirus-based vaccines. All approved options are live-attenuated formulations originally developed for smallpox [9]. While the protective efficacy of these vaccines is variable, their application is limited by safety profiles involving systemic adverse events and potential neurological complications [10]. Live-vaccinia based vaccines such as ACAM2000 have been associated with systemic adverse events (e.g., fever, malaise, myalgia) and serious neurological complications, including post-vaccinial encephalomyelitis (estimated incidence ~1/500 to 1/2000) [11]. Even non-replicating orthopoxvirus vaccines (e.g., MVA-BN/Imvanex) report neurological disorders (headache, dizziness, paraesthesia) in product-label data [12]. Such risks are particularly concerning for immunocompromised individuals, including people living with HIV [8,13,14]. The considerable global overlap between MPXV and HIV infections further underscores the clinical constraints of conventional vaccines against smallpox in this vulnerable subgroup [15,16].

Consequently, the development of next-generation monkeypox vaccines with enhanced safety and efficacy has emerged as a global priority. Several innovative platforms are currently under investigation, including mRNA vaccines, virus-like particles (VLPs), viral-vectored vaccines, and recombinant subunit vaccines [17,18,19,20,21]. Each platform offers unique advantages in terms of antigen design flexibility and capacity to elicit tailored immune responses. Among these, recombinant protein subunit vaccines represent a particularly promising alternative, characterized by their well-defined composition and absence of replicating material. This eliminates the risk of vaccine-induced infection, thereby eliminating the risk of replication and vaccine-induced infection. Such features make them especially appropriate for large-scale vaccination, including in immunocompromised populations. Furthermore, their favorable biochemical properties, including high purity, excellent stability, and scalable manufacturing, coupled with reduced cold-chain dependency, make them exceptionally suited for global immunization programs [17,22,23,24].

The immunogenic strength of subunit vaccines depends not only on the antigens but also critically on adjuvants. These components are essential for modulating the magnitude, quality, and breadth of adaptive immunity. Aluminum-based salts (alum), the most historically utilized adjuvants, function mainly by forming an antigen depot and promoting local inflammation [22,25,26]. As reported in previous studies, alum exhibits limited immunostimulatory capacity and predominantly drives Th2-biased humoral responses, while being relatively ineffective at inducing robust Th1-type cellular immunity [27,28]. Such a response is suboptimal for combating intracellular pathogens like MPXV, where Th1-driven mechanisms and cytotoxic T-cell activity are vital for viral clearance [29].

Novel nanoemulsion adjuvants have emerged as a potent and versatile alternative. These adjuvants activate NF-κB signaling at the injection site, triggering cytokine and chemokine release [30,31,32]. This inflammatory environment recruits monocytes, macrophages, and dendritic cells, which migrate to draining lymph nodes and enhance antigen uptake by antigen-presenting cells. With CD4^+^ T cell help, this process promotes robust B cell activation and antigen-specific antibody production [33,34]. When combined with PLGA, which provides sustained antigen release, squalene- and tocopherol-based nanoemulsions act synergistically to enhance immunostimulation and antigen persistence [35,36,37]. This integrated system facilitates efficient antigen processing and promotes potent, durable humoral and cellular immune responses [38].

A rational design for an MPXV subunit vaccine should account for the two distinct infectious forms of the virus: the intracellular mature virion (IMV), which supports viral persistence, and the extracellular enveloped virion (EEV), responsible for inter-host dissemination [35]. An effective immune response should target structural proteins from both virion types. Representative antigens, such as M1R and A29L from IMV, along with B6R and A35R from EEV, have been identified as promising vaccine candidates [18,39,40]. In light of this, we developed subunit formulations containing a proprietary nanoemulsion adjuvant and evaluated their immunogenicity and protective efficacy in a mouse model [38]. This comparative preclinical evaluation offers important evidence to advance the development of a safe and effective subunit vaccine against monkeypox.

## 2. Materials and Methods

### 2.1. Plasmids, Cells, and Viruses

The coding sequences of mpox clade II A29L, M1R, and B6R genes were codon-optimized and cloned into the BamHI and XbaI restriction sites of the pcDNA3.1 expression plasmid. To facilitate protein secretion, the human IL-6 signal peptide was fused to the 5′ end of each gene, while a 8× His tag was added to the 3′ end for subsequent Ni-NTA affinity purification.

BSC40 and BHK21 cells were maintained in Dulbecco’s modified Eagle’s medium (DMEM) supplemented with 10% fetal bovine serum (FBS) at 37 °C under general conditions. MPXV clade IIb strain NMU01 was isolated from an mpox patient in this laboratory in 2023. Vaccinia virus Western Reserve strain (VACV-WR) was kindly provided by Prof. Ke Zhang (Shanghai Institute of Immunity and Infection, Chinese Academy of Sciences). Viruses were propagated in BHK21 cells in DMEM with 2% heat-inactivated fetal bovine serum (FBS), and 100 U/mL penicillin and 100 µg/mL streptomycin. After 48 h, cells were harvested by centrifugation at 1000 rpm for 5 min and resuspended in PBS. The cell suspension was subjected to three freeze–thaw cycles to release intracellular mature virion (IMV) particles, followed by centrifugation at 5000 rpm for 10 min to remove cell debris. The clarified supernatants were aliquoted and stored at −80 °C. Viral titers were determined in Vero cells by plaque assay.

### 2.2. Protein Expression and Purification

Suspension-adapted 293F cells were seeded at a density of 0.3 × 10^6^ cells/mL in 10 mL of fresh FreeStyle™ 293 Expression Medium (Thermo Fisher Scientific, Grand Island, NY, USA). The cells were cultured in a shaking flask placed in an incubator shaker maintained at 37 °C, 5% CO_2_, and 120 rpm. When the cell density reached approximately 3 × 10^6^ cells/mL, the culture was diluted with fresh medium and incubated overnight. Plasmid transfection was then performed using polyethylenimine (PEI) as the transfection reagent, with a plasmid-to-PEI mass ratio of 1:3. The plasmid and PEI were first mixed in a 1.5 mL microcentrifuge tube and incubated at room temperature for 15 min. The mixture was then added dropwise into the cell suspension with gentle swirling to ensure even distribution. After transfection, the flask was returned to the shaker and cultured for an additional 3–4 days.

The culture was subsequently centrifuged at 3000× *g* for 10 min at 4 °C to remove the cells, and the supernatant was collected. Soluble proteins in the supernatant were purified through a series of steps including ultrafiltration, nickel-affinity chromatography, and desalting. The purified recombinant proteins were quantified using the BCA assay (Thermo Fisher Scientific), and their purity was verified by Coomassie blue staining.

### 2.3. Ethics Statement and Biosafety Compliance

All animal procedures were performed in full accordance with the Chinese Regulations for the Administration of Laboratory Animals and the Laboratory Animal Requirements for Environment and Housing Facilities (GB 14925-2010). Experimental protocols were reviewed and approved by the Animal Experiment Committee of Naval Medical University, China.

All experiments involving infectious orthopoxviruses were conducted in the accredited biosafety facilities of Naval Medical University. Work with live monkeypox virus (MPXV) was performed in Biosafety Level 3 (BSL-3) laboratories, whereas experiments involving vaccinia virus Western Reserve (VACV-WR) were conducted in Biosafety Level 2 (BSL-2) laboratories.

### 2.4. Nanoemulsion Adjuvant Preparation

A nanoemulsion adjuvant was prepared as previously described [38]. Briefly, 30 mg squalene, 23.7 mg tocopherol, and 10 mg Tween 80 were emulsified with 1 mL PBS containing 5 mg PLGA using probe sonication. The resulting formulation displayed a mean droplet diameter below 200 nm, a narrow size distribution (PDI < 0.2), and a zeta potential of approximately −5 mV. These physicochemical parameters remained stable for at least 15 days at 4 °C.

### 2.5. Mouse Immunization and Challenge

For vaccination, the nanoemulsion was gently shaken, mixed with a predetermined quantity of protein antigen, and then incubated at room temperature for 15 min. Female BALB/c mice, 6–8 weeks old, were used for immunization and virus challenge. Animals were randomly allocated into experimental groups using a random number–based assignment method. Each mouse was immunized intramuscularly with a 100 μL vaccine formulation containing a total of 10 μg protein. For single-antigen groups, 10 μg of recombinant protein was dissolved in 50 μL PBS and mixed with 50 μL nanoemulsion adjuvant (final 50% *v*/*v*). For the dual-antigen (A29L + B6R) groups, 5 μg of each antigen (total 10 μg) was dissolved together in 50 μL PBS, followed by addition of 50 μL nanoemulsion adjuvant or 50 μL aluminum hydroxide adjuvant (containing 100 μg alum), yielding a final injection volume of 100 μL for all formulations. Two immunizations were administered 3 weeks apart. Three weeks after the booster dose (day 42), mice were anesthetized with isoflurane inhalation and intranasally challenged with 50 μL of viral suspension containing either MPXV (1 × 10^7^ PFU) or vaccinia virus WR (1 × 10^5^ PFU). The inoculum was delivered dropwise onto the nostrils using a pipette, allowing the mice to inhale the suspension spontaneously under light anesthesia. Mice were monitored daily for changes in body weight and survival rates. At 5 days post-infection (dpi), a subset of mice was humanely euthanized to collect tissues (lungs and spleens) for viral load quantification and histopathological analysis. Investigators performing viral load measurements and histopathological evaluations were blinded to group allocation. The remaining mice were maintained for longitudinal survival analysis over a total period of 14 days.

### 2.6. Serum Antibody Analysis by ELISA

Mouse serum antibodies were measured using enzyme-linked immunosorbent assay (ELISA). Briefly, 96-well immunoassay plates were coated overnight at 4 °C with 1 μg/mL of recombinant MPXV A29L, B6R, or M1R protein. After blocking with a blocking buffer, the plates were incubated with serially diluted serum samples for 2 h at room temperature. Following a washing step, a horseradish peroxidase (HRP)-conjugated goat anti-mouse IgG secondary antibody (Thermo Fisher Scientific) was added and incubated for 1 h at 37 °C. The plates were then washed again, and 100 µL of 3,3′,5,5′-tetramethylbenzidine (TMB) substrate solution (Thermo Fisher Scientific, USA) was added to each well. The colorimetric reaction was allowed to develop for 20 min at room temperature and stopped with 50 μL of 2 M H_2_SO_4_. The endpoint titer was defined as the reciprocal of the highest serum dilution that yielded an absorbance value at 450 nm at least 2.1 times greater than that of the negative control sera.

### 2.7. Neutralizing Antibody Measurement

The neutralizing activity of immune mouse sera was measured using a plaque reduction neutralization test (PRNT). Serum samples were serially diluted twofold starting at 1:100 and mixed with an equal volume of MPXV or VACV-WR suspension prepared in DMEM supplemented with 2% fetal bovine serum (FBS). The mixtures were incubated at 37 °C for 1 h to allow neutralization, then inoculated onto BSC-40 cell monolayers in 96-well plates and incubated at 37 °C for 48 h. After incubation, cells were fixed with 4% paraformaldehyde and stained with 0.5% crystal violet to visualize infection foci. The neutralization titer (NT_50_) was defined as the highest serum dilution that resulted in a 50% reduction in the number of infection foci compared with the virus control wells.

### 2.8. Enzyme-Linked ImmunoSpot (ELISpot) Assay

T cell-mediated immune responses in mice were evaluated using an interferon-gamma (IFN-γ) ELISpot kit (Dakewe Biotech Co., Ltd., Shenzhen, China). Spleens were harvested and single-cell suspensions were prepared 14 days after the secondary immunization. ELISpot plates pre-coated with an IFN-γ monoclonal antibody were activated by adding 200 µL of serum-free ELISpot medium (Dakewe, China) per well and incubating for 5–10 min at room temperature. Subsequently, splenocytes (2 × 10^5^ per well) were seeded and stimulated with 60 μg/mL of MPXV proteins (A29L, B6R, or M1R) for 36 h at 37 °C in a 5% CO_2_ atmosphere. Following stimulation, cells were removed, and IFN-γ-secreting cells were detected using a biotinylated detection antibody and streptavidin-HRP. Spots were developed using a corresponding substrate and enumerated automatically with an S6 Ultra ImmunoSpot Reader (Cellular Technology Limited, Shaker Heights, OH, USA.). Data were analyzed using ImmunoSpot software (version 5.1.36, Cellular Technology Ltd.). The frequency of antigen-specific T cell responses was expressed as the number of spot-forming cells (SFC) per well.

### 2.9. Viral Load Detection by Real-Time Quantitative PCR (qPCR)

Viral DNA was extracted from lung tissue samples using the MagaBio Plus Virus DNA/RNA Purification Kit III (BIOER, BSC86S1E) according to the manufacturer’s instructions. The viral DNA loads of MPXV and vaccinia virus (VACV-WR) in lung tissues were quantified by real-time quantitative PCR (qPCR) using TB Green^®^ Premix Ex Taq™ II (Takara Bio Inc., Shiga, Japan.) on a QuantStudio 3 Real-Time PCR System (Applied Biosystems, Thermo Fisher Scientific, Foster City, CA, USA). The following primer sequences were used for detection: MPXV/VACV-WR: Forward: 5′-GGA AAA TGT AAA GAC AAC GAA TAC AG-3′, Reverse: 5′-GCT ATC ACA TAA TCT GGA AGC GTA-3′; Gapdh (genomic reference gene): Forward: 5′-AGC CAT CAG CTA TGC ACG TA-3′, Reverse: 5′-GGA CTT TCT AGG GTG GGA ACA G-3′. For MPXV, a standard curve was generated using a plasmid of known copy number, and viral DNA copies in samples were calculated based on Ct values. For VACV-WR, viral Ct values were normalized to the endogenous control Gapdh, and relative viral DNA levels were determined using the 2^^−ΔΔCt^ method. The viral DNA load in the lung tissues of mice immunized with nanoemulsion adjuvant alone was set as the baseline (value = 1).

### 2.10. Statistical Analysis

Statistical analyses were conducted using GraphPad Prism 8.0 (GraphPad Software). Between-group comparisons were performed using either unpaired or paired two-tailed Student’s *t*-tests as appropriate, while multiple group comparisons were analyzed by one-way ANOVA followed by Tukey’s post hoc test. Statistical significance was defined as *p* < 0.05, with the following notation: * *p* < 0.05, ** *p* < 0.01, and *** *p* < 0.001; ns (not significant) for *p* ≥ 0.05.

## 3. Results

### 3.1. Expression, Purification, and Characterization of MPXV Proteins

**Figure 1:**
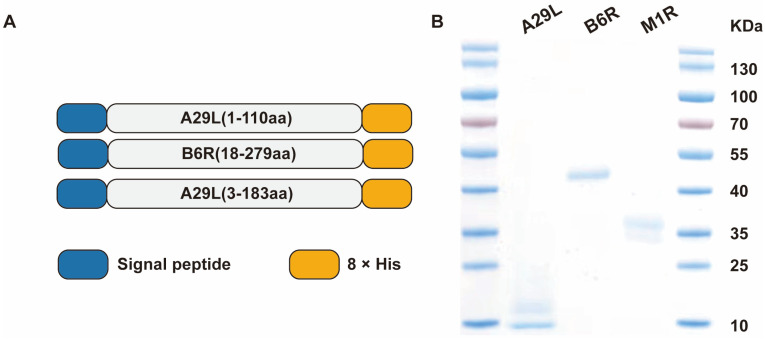
Expression and Characterization of MPXV A29L, B6R, and M1R Proteins. (**A**) Gene constructs of A29L, B6R and M1R. (**B**) SDS-PAGE analysis of the purified proteins, visualized by Coomassie blue staining.

The A29L, M1R, and B6R genes of mpox clade II were codon-optimized and synthesized. To facilitate recombinant protein secretion and purification, the human IL-6 signal peptide and a 8× His tag sequence were incorporated at the N- and C-termini, respectively (Figure 1A). Each resulting sequence was cloned into the pcDNA3.1 mammalian expression vector. For protein production, the respective plasmids were transfected into FreeStyle™ 293-F cells. Each recombinant protein was then purified from the culture supernatant using Ni-NTA affinity chromatography. Following purification, the proteins were desalted, sterile-filtered (0.22 µm), and analyzed by SDS-PAGE. Each sample showed a single, predominant band at the expected molecular weight, confirming high purity, which was quantified to be greater than 90% (Figure 1B). Furthermore, endotoxin levels in the final protein preparations were determined by the LAL method to be below 1.0 EU per μg of protein.

### 3.2. Immunogenicity of Nanoemulsion Adjuvanted Subunit Vaccines in Mice

**Figure 2:**
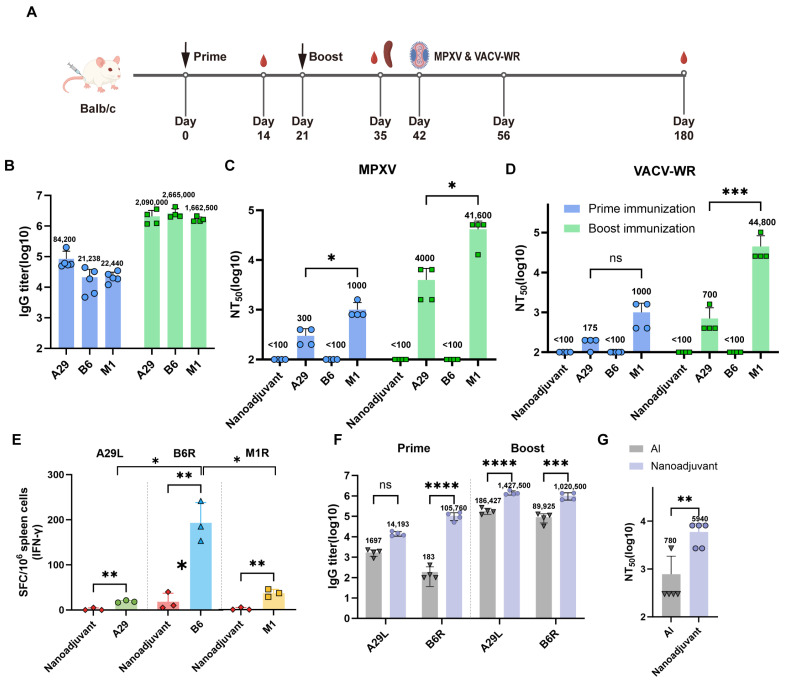
Evaluation of immune responses to MPXV subunit vaccines. (**A**) Schematic diagram of the mouse immunization and challenge schedule. Six- to eight-week-old female BALB/c mice were immunized by intramuscular (i.m.) injection with 100 μL of vaccine formulations. Immunizations were administered on day 0 and day 21. At week 3 post-boost (day 42), mice were challenged via intranasal instillation with either MPXV (1 × 10^7^ PFU/mouse) or VACV-WR (1 × 10^5^ PFU/mouse) to evaluate vaccine-induced protection. (**B**) Peripheral blood serum was collected on day 14 post-prime and day 14 post-boost (day 35). Antigen-specific IgG endpoint titers against A29L, B6R, and M1R were determined by ELISA (*n* = 4 per group). (**C**,**D**) Using serum from the same time points, neutralizing antibody titers against MPXV (**C**) and VACV-WR (**D**) were assessed by a plaque reduction neutralization test (PRNT) and are expressed as NT_50_ (*n* = 4 per group). (**E**) Splenocytes were harvested on day 14 post-boost and restimulated ex vivo with the corresponding recombinant protein (A29L, B6R, M1R) or PBS. Antigen-specific cellular immune responses were measured by IFN-γ ELISpot and are presented as spot-forming cells (SFCs) per 10^6^ cells (*n* = 3 per group). (**F**,**G**) Comparison of the adjuvant effects on immune responses. Serum samples from the alum and nanoemulsion adjuvant groups (both with A29 + B6) collected on day 35 were analyzed. (**F**) A29L- and B6R-specific IgG titers were measured by ELISA (*n* = 4 per group). (**G**) Neutralizing antibody titers against MPXV were determined by PRNT (*n* = 5 per group). * *p* < 0.05; ** *p* < 0.01; *** *p* < 0.001; **** *p* < 0.0001.

To evaluate the immunogenicity of individual antigens, BALB/c mice were immunized intramuscularly twice at a 3-week interval with monkeypox virus proteins (A29L, B6R, or M1R), each formulated with a nanoemulsion adjuvant at a 1:1 volume ratio (A29, B6, or M1 groups). Each mouse received a 10 µg dose of the respective antigen. Control groups received PBS mixed with adjuvant. Serum samples were collected two weeks after each immunization for subsequent antibody analysis (Figure 2A).

ELISA results indicated that all three recombinant proteins elicited detectable IgG antibodies after the prime immunization. A booster elicited a 10–20-fold rise in titers across all groups. The resulting endpoint titers reached comparable levels across all antigen groups (*n* = 4–5 per group, Figure 2B).

The neutralizing activity of immune sera collected after the booster immunization against MPXV and Vaccinia virus (VACV) was assessed using a micronutralization assay. The results showed that M1R immune serum yielded the highest neutralizing titers against both MPXV and VACV, with NT50 values of 41,600 and 44,800, respectively. The NT50 for VACV was 1.07-fold higher than that for MPXV. A29L immune serum also exhibited significant, though comparatively weaker, neutralizing activity against both viruses compared to M1R serum, with NT50 values of 4000 and 700 for MPXV and VACV, respectively, representing a 5.71-fold difference. In contrast, the neutralizing titer of B6R immune serum was below the detection limit, indicating no significant neutralization was detected even at a 100-fold dilution (*n* = 4 per group, Figure 2C,D). This is likely attributable to the predominance of the MV form of the virus in the cell culture used for the assay.

A Th1-type cellular immune response, characterized by IFN-γ secretion, plays a critical role in viral clearance. Splenocytes were isolated from mice in each group on day 14 post-booster immunization, and IFN-γ secretion was evaluated by ELISpot to assess the cellular immune response induced by the recombinant proteins. The number of IFN-γ-positive cells in the B6 immunization group was significantly higher than those in the A29 and M1 groups (*n* = 3 per group, Figure 2E and Figure S1), suggesting that B6R most effectively elicits a Th1-type cellular immune response.

To compare the efficacy of the nanoemulsion adjuvant versus aluminum hydroxide (Al) adjuvant in formulating subunit antigens, immunization groups were established using either nano-emulsion or Al adjuvant combined with a dual-antigen formulation of A29L and B6R (representing IMV and EV antigens, respectively), with the antigen dose reduced to 5 µg each. Results showed that after the first immunization, the B6R-specific antibody titer in the nano-emulsion group was 563.75-fold higher than that in the Al adjuvant group (*p* < 0.001), whereas the A29L-specific IgG titer was also 8.36-fold higher but the difference was not statistically significant (*p* = 0.6878). Sera from mice after the booster immunization showed that the nano-emulsion group had significantly higher levels of B6R-specific antibodies, A29L-specific IgG, and neutralizing antibodies compared to the Al adjuvant group (*n* = 4–5 per group, Figure 2F,G).

### 3.3. Protection Against Lethal MPXV Challenge

To assess the protective efficacy of the various vaccine formulations, mice were intranasally challenged with MPXV at a dose of 10^7^ PFU per mouse. Body weight changes and survival were monitored daily for 14 days post-challenge.

**Figure 3:**
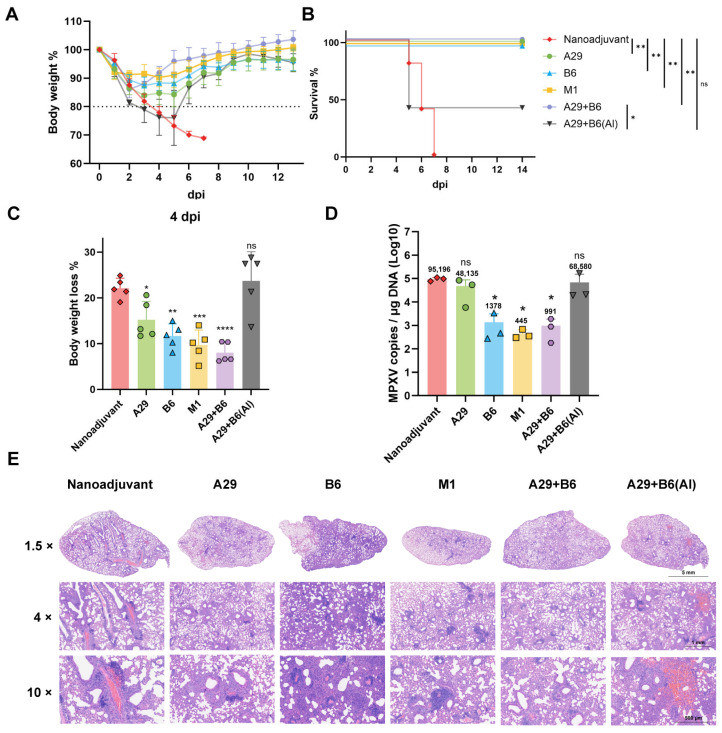
Protective efficacy of subunit vaccines against lethal MPXV challenge. Mice were challenged intranasally with 1 × 10^7^ PFU of MPXV 21 days after the booster immunization. Body weight changes and survival were monitored daily for 14 days. Lung tissues were collected on day 4 post-infection for quantification of MPXV viral load (by qPCR) and histological analysis (by H&E staining). (**A**) Body weight changes presented as the mean percentage of initial weight (*n* = 5 per group). (**B**) Survival rates (*n* = 5 per group). (**C**) Percentage of body weight loss on day 4 post-infection (*n* = 5 per group). (**D**) MPXV gene copies in lung tissue measured by qPCR (*n* = 3 per group). (**E**) Representative H&E-stained lung sections. Data are presented as mean ± SEM. Survival curves were analyzed using the log-rank (Mantel–Cox) test with multiple-group comparison. Statistical significance was determined by one-way ANOVA (* *p* < 0.05; ** *p* < 0.01; *** *p* < 0.001; **** *p* < 0.0001).

Mice in the nano-emulsion adjuvant control group exhibited progressive weight loss, reaching approximately 20% by day 4. The dual-antigen (A29 + B6) Al adjuvant group demonstrated more pronounced weight loss during the initial 1–4 days post-challenge; however, a subset of mice in this group began to regain weight starting on day 5. On day 4 post-challenge, mice immunized with A29L showed approximately 15% weight loss, whereas those in the B6 group lost about 12%. In contrast, both the M1 group and the dual-antigen (A29 + B6) nano-emulsion group were effectively protected, exhibiting minimal weight loss that remained below 10% throughout the observation period (*n* = 5 per group, Figure 3A,C). Survival analysis revealed that all mice in the nano-emulsion groups survived the challenge, whereas mortality was observed in the nano-adjuvant control and A29 + B6 (Al) groups, confirming the superior protective efficacy of the nano-emulsion formulation (*n* = 5 per group, Figure 3B).

On day 5 post-challenge, three mice per group were euthanized for lung collection to quantify viral load (copies per µg lung tissue DNA) and to perform histopathological analysis (*n* = 3 per group, Figure 3D). The nano-emulsion control group exhibited a high pulmonary viral load of approximately 95,196 copies/μg DNA. In contrast, the M1R-immunized group showed the lowest viral burden, at approximately 445 copies/μg DNA, representing a 213.9-fold reduction compared to the control. The dual-antigen (A29 + B6) nano-emulsion and B6R groups also displayed significantly lower viral loads than the control group (*p* < 0.05). Conversely, both the A29 group and the dual-antigen (A29 + B6) Al adjuvant group sustained high viral loads (approximately 48,135 and 68,580 copies/μg DNA, respectively), which were not statistically different from the nano-emulsion control.

Histopathological examination of lung tissues from the nano-emulsion control group revealed severe alveolar architecture disruption, diffuse inflammatory cell infiltration, significant interstitial thickening, and marked hemorrhage (*n* = 3 per group, Figure 3E). The dual-antigen (A29 + B6) Al adjuvant group showed moderately attenuated but still substantial pathology. Mice in the A29 group developed moderate lesions characterized by focal inflammatory cell aggregates. The B6 group exhibited considerable inflammation and interstitial thickening, though without significant hemorrhage. In comparison, lung tissues from the M1 group and the dual-antigen (A29 + B6) nano-emulsion group displayed largely preserved alveolar structure, accompanied only by mild inflammatory infiltration and an absence of notable hemorrhage or substantive tissue damage.

### 3.4. Cross-Protective Against Lethal VACV-WR Challenge

**Figure 4:**
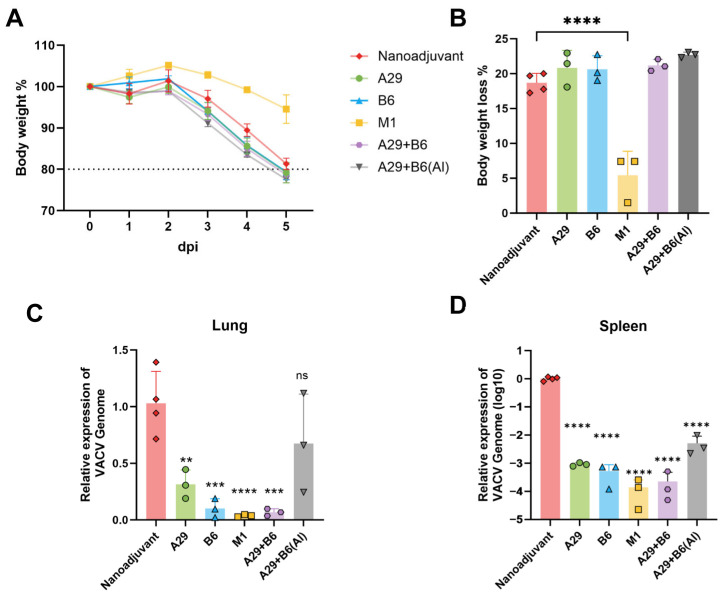
Cross-protective efficacy of subunit vaccines against lethal VACV-WR challenge. Mice were challenged intranasally with 1 × 10^5^ PFU of VACV-WR 21 days after the booster immunization (*n* = 3 for antigen immunization per group, *n* = 4 for nanoemulsion adjuvant control group.). Body weight was monitored daily for 5 days post-infection. On day 5, lung and spleen tissues were collected to determine viral load by quantitative real-time PCR. Relative viral DNA content was calculated using the 2^^−ΔΔCt^ method, with the level in the nanoemulsion adjuvant control group set as 1. (**A**) Body weight changes from day 0 to 5 post-infection. (**B**) Percentage of body weight loss on day 5 post-infection. (**C**,**D**) Relative viral load in lung (**C**) and spleen (**D**) tissues. Data are presented as mean ± SEM. Statistical significance was determined by one-way ANOVA (** *p* < 0.01; *** *p* < 0.001; **** *p* < 0.0001).

Given the close antigenic relationship between MPXV and vaccinia virus [36,37], we further evaluated the cross-protective efficacy of the individual MPXV subunit vaccines against vaccinia virus. Three weeks after the booster immunization, mice were intranasally challenged with VACV-WR at a dose of 10^5^ PFU per mouse. Body weight was monitored daily for five days, after which all animals were euthanized for quantification of viral loads in the lungs and spleens. By day 5 post-challenge, control group mice exhibited approximately 20% body weight loss. In contrast, mice immunized with M1R displayed minimal weight loss (<10%), whereas no significant difference in weight change was observed in the other immunization groups compared with the control (*n* = 3–4 per group, Figure 4A,B).

Viral load was assessed in lungs and spleens harvested 5 days post-challenge. In lungs, all but the dual-antigen (A29 + B6) Alum group had significantly lower viral DNA than the control, with the M1R group exhibiting the most markedly reduced viral DNA levels (*n* = 3–4 per group, Figure 4C). In spleens, viral loads were lower than in lungs across all groups, and all vaccine groups showed significant suppression compared to the control (*n* = 3–4 per group, Figure 4D).

### 3.5. Long-Term Humoral Immune Responses Induced by Nanoemulsion Adjuvanted Subunit Vaccines

**Figure 5:**
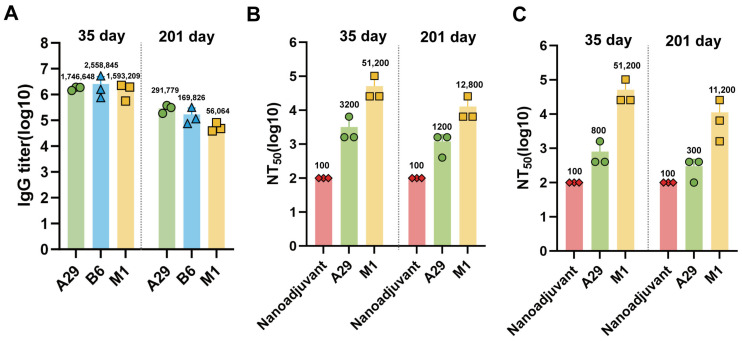
Durability of vaccine-elicited antibody responses. Serum samples were collected at two weeks (Day 35) and six months (Day 201) after the booster immunization to evaluate the persistence of humoral immunity. (**A**) IgG antibody titers against MPXV proteins were measured by ELISA at the indicated time points (*n*= 3 per group). (**B**,**C**) Neutralizing antibody titers against MPXV (**B**) and VACV-WR (**C**) in the serum were determined by a plaque reduction neutralization test (PRNT) and are expressed as NT_50_ (*n* = 3 per group).

To evaluate the persistence of humoral immune responses following immunization with nanoemulsion adjuvanted subunit vaccines, mouse sera were collected six months after the booster immunization to assess specific IgG antibody levels and their neutralizing activity. ELISA results demonstrated that IgG titers decreased from 1.75 × 10^6^ to 2.92 × 10^5^ in the A29L group (~6-fold), from 2.56 × 10^6^ to 1.70 × 10^5^ in the B6R group (~15-fold), and from 1.59 × 10^6^ to 5.61 × 10^4^ in the M1R group (~28-fold) between day 35 and day 201; however, none of these changes reached statistical significance (*n* = 3 per group, Figure 5A). Similarly, neutralizing titers showed only partial decreases but remained readily detectable at the late time point. For MPXV, NT_50_ titers decreased from 3200 to 1200 in the A29L group (~2.7-fold) and from 51,200 to 12,800 in the M1R group (~4-fold) (*n* = 3 per group, Figure 5B). For VACV-WR, NT_50_ titers decreased from 800 to 300 in the A29L group (~2.7-fold) and from 51,200 to 11,200 in the M1R group (~4.6-fold) (*n* = 3 per group, Figure 5C). Notably, M1R-immunized mice retained the highest neutralizing activity at both time points. These findings indicate that the nanoemulsion-adjuvanted subunit vaccines can induce durable humoral immune responses.

## 4. Discussion

The persistent global mpox outbreak since 2022 highlights an urgent need for effective vaccines specifically targeting MPXV. Recombinant protein vaccines are a promising candidate due to their established safety profile, scalable production, and stability, all of which favor global accessibility [41,42,43]. A common strategy to address the complex antigenicity of orthopoxviruses is to combine multiple immunogens [19]. However, this increases manufacturing complexity and cost, potentially limiting access in resource-limited, high-burden regions such as Africa [44,45]. Therefore, identifying a minimal set of highly effective antigens is crucial for developing safe, efficacious, and widely deployable mpox vaccines. Adjuvants are essential components of recombinant subunit vaccines, making the selection of an appropriate adjuvant critical for inducing protective immunity.

In this study, we assessed the immunogenicity and protective efficacy of a novel nanoemulsion adjuvant, formulated with three key MPXV structural proteins (A29L, B6R, and M1R), in a mouse model. Mice immunized with a prime-boost regimen of the three individual antigens (in equal amounts) combined with the nanoemulsion adjuvant elicited comparable serum IgG levels against each antigen. However, in vitro neutralization assays against both MPXV and VACV-WR revealed that B6R immune serum showed no significant neutralizing activity. This is likely because B6 is an EEV membrane protein, whereas IMV particles overwhelmingly dominate in virions obtained from cell culture [46,47]. Consistent with this notion, A29L and M1R, as IMV membrane proteins, induced serum antibodies with potent neutralizing activity. Notably, M1R antiserum exhibited stronger neutralization than A29L antiserum, particularly against VACV-WR. This finding not only suggests M1R as a preferred vaccine antigen but also implies that despite high genomic homology among MPXV, VACA and smallpox virus, key neutralizing epitopes may differ significantly. This highlights the necessity of developing vaccines specifically tailored against MPXV for optimal protection.

A Th1-type cellular immune response, typified by IFN-γ secretion, is crucial for clearing intracellular viral infections and is a desired feature of antiviral vaccines. Our ELISpot analyses revealed that splenocytes from B6R-immunized mice mounted a robust IFN-γ response. While A29L and M1R also induced significant T-cell responses compared to the adjuvant control, they were markedly lower than that elicited by B6R. This observation is consistent with a previous report, reinforcing the role of B6R in driving Th1-type immunity [48].

To further evaluate the utility of our nanoemulsion adjuvant, we employed a bivalent vaccine strategy combining A29L (IMV) and B6R (EEV) antigens. This combination was selected prior to our recognition of M1’s superior neutralizing antibody induction. The nanoemulsion-adjuvanted bivalent vaccine induced significantly higher antigen-specific IgG against both antigens and neutralizing antibody titers than the aluminum hydroxide-adjuvanted formulation.

Upon intranasal MPXV challenge, all three monovalent vaccines conferred complete (100%) survival. However, post-challenge body weight changes and lung viral DNA loads on day 5 indicated that M1 provided the strongest protection, followed by B6, with A29 being comparatively less effective. The partial protection observed with B6R may be attributable to its ability to induce robust Th1-type T-cell responses; however, this immunological profile alone was insufficient to achieve the level of viral clearance elicited by M1R [40]. The nanoemulsion-adjuvanted bivalent vaccine also demonstrated significantly enhanced protection compared to its aluminum hydroxide counterpart, reinforcing the advantage of this adjuvant system.

In a VACV-WR lethal challenge model, only M1 immunization conferred significant protection as measured by body weight preservation. The limited protection in other groups, including the A29 + B6 bivalent vaccine, further underscores the central role of M1 as a highly effective immunogen. Viral DNA quantification in lungs and spleen on day 5 post-challenge revealed that all three antigens significantly suppressed viral replication. Consistent with MPXV challenge data, M1 again exhibited the strongest inhibition of viral load in the lungs. For the bivalent vaccine, the aluminum hydroxide formulation was significantly less effective at suppressing viral replication than the nanoemulsion formulation.

At the late time point, antigen-specific IgG and neutralizing antibody titers remained readily detectable six months after immunization, indicating durable humoral responses. However, only serological and neutralization assays were performed at this stage, and no additional challenge experiment was conducted. Therefore, any inference regarding long-term protection is based on serological correlates rather than direct functional validation.

One noteworthy observation in our study concerns the lung pathology in B6R-immunized mice after MPXV challenge (Figure S2). Although B6R vaccination achieved substantial viral clearance—second only to M1R—the associated pulmonary inflammation was more pronounced than in the nanoemulsion adjuvant control group. This dissociation between viral clearance and tissue inflammation suggests that B6R-induced immune responses may not be uniformly protective at the tissue level. Given that B6R elicited the strongest IFN-γ responses among the three antigens, T-cell-mediated cytotoxicity or related effector mechanisms could contribute to this inflammation; however, our current data do not establish a causal relationship, and this remains a hypothesis. Additional pathways, such as antibody-dependent cellular cytotoxicity (ADCC), also cannot be excluded. Future studies incorporating multicytokine profiling (e.g., IL-6, TNF-α) and CD4^+^/CD8^+^ T-cell subset analyses will be required to clarify the immunological drivers of the observed pathology and fully assess the safety profile of B6R as a vaccine antigen.

The present study was conducted in young, immunocompetent female BALB/c mice, which represents a widely used model for preclinical vaccine evaluation but does not capture the immunological characteristics of high-risk human populations. Individuals with advanced HIV infection, transplant recipients under immunosuppressive therapy, and elderly populations may respond differently to subunit vaccination. Therefore, caution is warranted when extrapolating the protective efficacy observed here to immunocompromised or clinically vulnerable groups. Future studies will include evaluation in aged or immunocompromised mouse models, and ultimately in nonhuman primates, to better assess the translational potential of these vaccine candidates in populations most affected by mpox.

In conclusion, our study demonstrates that the nanoemulsion adjuvant, when combined with any of the three MPXV antigens, induces robust immune responses and significant protection in mice. Its superior performance over aluminum hydroxide in the bivalent formulation highlights its strong application potential. Furthermore, we identified the M1 antigen as a promising antigen candidate due to its ability to elicit potent neutralizing antibodies and confer excellent protection. While B6R shows an advantage in inducing Th1-type cellular immunity and provides good protection, its potential safety concerns necessitate further careful evaluation.

## Data Availability

The original contributions presented in the study are included in the article, further inquiries can be directed to the corresponding author.

## Supplementary Materials

The following supporting information can be downloaded at: https://www.mdpi.com/article/10.3390/pathogens14121293/s1, Figure S1: Representative IFN-γ ELISpot images from splenocytes collected on day 14 post-boost; Figure S2: Lung histopathology scoring following MPXV challenge.

## References

[1] Lum F.-M., Torres-Ruesta A., Tay M.Z., Lin R.T.P., Lye D.C., Rénia L., Ng L.F.P. (2022). Monkeypox: Disease Epidemiology, Host Immunity and Clinical Interventions. Nat. Rev. Immunol..

[2] Adetifa I., Muyembe J.-J., Bausch D.G., Heymann D.L. (2023). Mpox Neglect and the Smallpox Niche: A Problem for Africa, a Problem for the World. Lancet.

[3] Thornhill J.P., Barkati S., Walmsley S., Rockstroh J., Antinori A., Harrison L.B., Palich R., Nori A., Reeves I., Habibi M.S. (2022). Monkeypox Virus Infection in Humans across 16 Countries—April–June 2022. N. Engl. J. Med..

[4] Zumla A., Valdoleiros S.R., Haider N., Asogun D., Ntoumi F., Petersen E., Kock R. (2022). Monkeypox Outbreaks Outside Endemic Regions: Scientific and Social Priorities. Lancet Infect. Dis..

[5] Sukhdeo S., Mishra S., Walmsley S. (2022). Human Monkeypox: A Comparison of the Characteristics of the New Epidemic to the Endemic Disease. BMC Infect. Dis..

[6] Epidemiological Update—Week 35/2024: Mpox Due to Monkeypox Virus Clade I. https://www.ecdc.europa.eu/en/news-events/mpox-epidemiological-update-monkeypox-2-september-2024.

[7] Ahmed S.K., Ahmed Rashad E.A., Mohamed M.G., Ravi R.K., Essa R.A., Abdulqadir S.O., Khdir A.A. (2022). The Global Human Monkeypox Outbreak in 2022: An Overview. Int. J. Surg..

[8] Rao A.K., Petersen B.W., Whitehill F., Razeq J.H., Isaacs S.N., Merchlinsky M.J., Campos-Outcalt D., Morgan R.L., Damon I., Sánchez P.J. (2022). Use of JYNNEOS (Smallpox and Monkeypox Vaccine, Live, Nonreplicating) for Preexposure Vaccination of Persons at Risk for Occupational Exposure to Orthopoxviruses: Recommendations of the Advisory Committee on Immunization Practices—United States, 2022. MMWR Morb. Mortal. Wkly. Rep..

[9] Lozano J.M., Muller S. (2023). Monkeypox: Potential Vaccine Development Strategies. Trends Pharmacol. Sci..

[10] Wahid M., Mandal R.K., Sikander M., Khan M.R., Haque S., Nagda N., Ahmad F., Rodriguez-Morales A.J. (2025). Safety and Efficacy of Repurposed Smallpox Vaccines Against Mpox: A Critical Review of ACAM2000, JYNNEOS, and LC16. J. Epidemiol. Glob. Health.

[11] Billioux B.J., Mbaya O.T., Sejvar J., Nath A. (2022). Neurologic Complications of Smallpox and Monkeypox: A Review. JAMA Neurol..

[12] Farahat R.A., Shrestha A.B., Elsayed M., Memish Z.A. (2022). Monkeypox Vaccination: Does It Cause Neurologic and Psychiatric Manifestations?—Correspondence. Int. J. Surg..

[13] Greenberg R.N., Overton E.T., Haas D.W., Frank I., Goldman M., von Krempelhuber A., Virgin G., Bädeker N., Vollmar J., Chaplin P. (2013). Safety, Immunogenicity, and Surrogate Markers of Clinical Efficacy for Modified Vaccinia Ankara as a Smallpox Vaccine in HIV-Infected Subjects. J. Infect. Dis..

[14] Fang Y., Wang F., Jiang T., Duan J., Huang T., Liu H., Jia L., Jia H., Yan B., Zhang M. (2024). Human Mpox Co-Infection with Advanced HIV-1 and XDR-TB in a MSM Patient Previously Vaccinated against Smallpox: A Case Report. Biosaf. Health.

[15] Poland G.A., Kennedy R.B., Tosh P.K. (2022). Prevention of Monkeypox with Vaccines: A Rapid Review. Lancet Infect. Dis..

[16] Nakamura H., Yamamoto K. (2024). Mpox in People with HIV: A Narrative Review. HIV Med..

[17] Fang Z., Monteiro V.S., Renauer P.A., Shang X., Suzuki K., Ling X., Bai M., Xiang Y., Levchenko A., Booth C.J. (2023). Polyvalent mRNA Vaccination Elicited Potent Immune Response to Monkeypox Virus Surface Antigens. Cell Res..

[18] Kong T., Du P., Ma R., Wang H., Ma X., Lu J., Gao Z., Qi H., Li R., Zhang H. (2024). Single-Chain A35R-M1R-B6R Trivalent mRNA Vaccines Protect Mice against Both Mpox Virus and Vaccinia Virus. eBioMedicine.

[19] Garcia-Atutxa I., Mondragon-Teran P., Huerta-Saquero A., Villanueva-Flores F. (2024). Advancements in Monkeypox Vaccines Development: A Critical Review of Emerging Technologies. Front. Immunol..

[20] Belghith A.A., Cotter C.A., Ignacio M.A., Earl P.L., Hills R.A., Howarth M.R., Yee D.S., Brenchley J.M., Moss B. (2025). Mpox Multiprotein Virus-like Nanoparticle Vaccine Induces Neutralizing and Protective Antibodies in Mice and Non-Human Primates. Nat. Commun..

[21] Saadh M.J., Ghadimkhani T., Soltani N., Abbassioun A., Daniel Cosme Pecho R., Taha A., Jwad Kazem T., Yasamineh S., Gholizadeh O. (2023). Progress and Prospects on Vaccine Development against Monkeypox Infection. Microb. Pathog..

[22] Reed S.G., Orr M.T., Fox C.B. (2013). Key Roles of Adjuvants in Modern Vaccines. Nat. Med..

[23] Hou Y., Chen M., Bian Y., Zheng X., Tong R., Sun X. (2023). Advanced Subunit Vaccine Delivery Technologies: From Vaccine Cascade Obstacles to Design Strategies. Acta Pharm. Sin. B.

[24] Pollet J., Chen W.-H., Strych U. (2021). Recombinant Protein Vaccines, a Proven Approach against Coronavirus Pandemics. Adv. Drug Deliv. Rev..

[25] Marrack P., McKee A.S., Munks M.W. (2009). Towards an Understanding of the Adjuvant Action of Aluminium. Nat. Rev. Immunol..

[26] Awate S., Babiuk L.A., Mutwiri G. (2013). Mechanisms of Action of Adjuvants. Front. Immunol..

[27] Cao H., Yang S., Wang Y., Luan N., Yin X., Lin K., Liu C. (2021). An Established Th2-Oriented Response to an Alum-Adjuvanted SARS-CoV-2 Subunit Vaccine Is Not Reversible by Sequential Immunization with Nucleic Acid-Adjuvanted Th1-Oriented Subunit Vaccines. Vaccines.

[28] Turley J.L., Lavelle E.C. (2022). Resolving Adjuvant Mode of Action to Enhance Vaccine Efficacy. Curr. Opin. Immunol..

[29] De Gregorio E., D’Oro U., Wack A. (2009). Immunology of TLR-Independent Vaccine Adjuvants. Curr. Opin. Immunol..

[30] Zhao T., Cai Y., Jiang Y., He X., Wei Y., Yu Y., Tian X. (2023). Vaccine Adjuvants: Mechanisms and Platforms. Signal Transduct. Target. Ther..

[31] Pulendran B., Arunachalam P.S., O’hAgan D.T. (2021). Emerging Concepts in the Science of Vaccine Adjuvants. Nat. Rev. Drug Discov..

[32] Fox C.B., Baldwin S.L., Duthie M.S., Reed S.G., Vedvick T.S. (2011). Immunomodulatory and Physical Effects of Oil Composition in Vaccine Adjuvant Emulsions. Vaccine.

[33] Yoo Y.J., Kim S., Wickramasinghe A., Kim J., Song J., Kim Y.-I., Gil J., Noh Y.-W., Lee M.-H., Oh S.-S. (2025). Multiscale Dynamic Immunomodulation by a Nanoemulsified Trojan-TLR7/8 Adjuvant for Robust Protection against Heterologous Pandemic and Endemic Viruses. Cell. Mol. Immunol..

[34] Morel S., Didierlaurent A., Bourguignon P., Delhaye S., Baras B., Jacob V., Planty C., Elouahabi A., Harvengt P., Carlsen H. (2011). Adjuvant System AS03 Containing α-Tocopherol Modulates Innate Immune Response and Leads to Improved Adaptive Immunity. Vaccine.

[35] Cassaidy B.J., Moser B.A., Solanki A., Chen Q., Shen J., Gotsis K., Lockhart Z., Rutledge N., Rosenberger M.G., Dong Y. (2024). Immune Potentiation of PLGA Controlled-Release Vaccines for Improved Immunological Outcomes. ACS Omega.

[36] Yoshioka Y., Kobiyama K., Hayashi T., Onishi M., Yanagida Y., Nakagawa T., Hashimoto M., Nishinaka A., Hirose J., Asaoka Y. (2023). A-910823, a Squalene-Based Emulsion Adjuvant, Induces T Follicular Helper Cells and Humoral Immune Responses via α-Tocopherol Component. Front. Immunol..

[37] Li Z., Chen P., Qu A., Sun M., Xu L., Xu C., Hu S., Kuang H. (2025). Opportunities and Challenges for Nanomaterials as Vaccine Adjuvants. Small Methods.

[38] Long Y., Sun J., Song T.-Z., Liu T., Tang F., Zhang X., Ding L., Miao Y., Zhu W., Pan X. (2022). CoVac501, a Self-Adjuvanting Peptide Vaccine Conjugated with TLR7 Agonists, against SARS-CoV-2 Induces Protective Immunity. Cell Discov..

[39] Cheng X., Wang Y., Huang B., Bing J., Wang T., Han R., Huo S., Sun S., Zhao L., Shu C. (2025). Rational Mpox Vaccine Design: Immunogenicity and Protective Effect of Individual and Multicomponent Proteins in Mice. Emerg. Microbes Infect..

[40] Qu Y., Tai W., Ma E., Jiang Q., Fan M., Xiao W., Tian C., Liu Y., Liu J., Wang X. (2025). Generation and Characterization of Neutralizing Antibodies against M1R and B6R Proteins of Monkeypox Virus. Nat. Commun..

[41] Interim Recommendations for the Use of Protein Subunit COVID-19 Vaccines. https://www.who.int/publications/i/item/WHO-2019-nCoV-vaccines-SAGE_recommendation-protein_subunit-2023.1.

[42] de Pinho Favaro M.T., Atienza-Garriga J., Martínez-Torró C., Parladé E., Vázquez E., Corchero J.L., Ferrer-Miralles N., Villaverde A. (2022). Recombinant Vaccines in 2022: A Perspective from the Cell Factory. Microb. Cell. Factories.

[43] CDC Storage and Handling of Immunobiologics. https://www.cdc.gov/vaccines/hcp/imz-best-practices/storage-handling-immunobiologics.html.

[44] Protopapas K., Dimopoulou D., Kalesis N., Akinosoglou K., Moschopoulos C.D. (2024). Mpox and Lessons Learned in the Light of the Recent Outbreak: A Narrative Review. Viruses.

[45] Zeng J., Li Y., Jiang L., Luo L., Wang Y., Wang H., Han X., Zhao J., Gu G., Fang M. (2023). Mpox Multi-Antigen mRNA Vaccine Candidates by a Simplified Manufacturing Strategy Afford Efficient Protection against Lethal Orthopoxvirus Challenge. Emerg. Microbes Infect..

[46] Tan M., Zhang R., Shen T., Li A., Hou X., Zhang Y., Wang T., Zhang B., Sun P., Gong X. (2024). Systematic Evaluation of the Induction of Efficient Neutralizing Antibodies by Recombinant Multicomponent Subunit Vaccines against Monkeypox Virus. Vaccine.

[47] Jones-Trower A., Garcia A., Meseda C.A., He Y., Weiss C., Kumar A., Weir J.P., Merchlinsky M. (2005). Identification and Preliminary Characterization of Vaccinia Virus (Dryvax) Antigens Recognized by Vaccinia Immune Globulin. Virology.

[48] Li J., Li X., Dong J., Wei J., Guo X., Wang G., Xu M., Zhao A. (2024). Enhanced Immune Responses in Mice by Combining the Mpox Virus B6R-Protein and Aluminum Hydroxide-CpG Vaccine Adjuvants. Vaccines.

