# Supplementary material for: Immune Responses and Protective Efficacy of Nanoemulsion-Adjuvanted Monkeypox Virus Recombinant Vaccines Against Lethal Challenge in Mice
Source: Pathogens. 2025 Dec 16;14(12):1293. doi: 10.3390/pathogens14121293 (PMC12735760; doi:10.3390/pathogens14121293)
Supplement: Supplementary file 1 [file pathogens-14-01293-s001.zip › pathogens-3990076-supplementary.pdf]

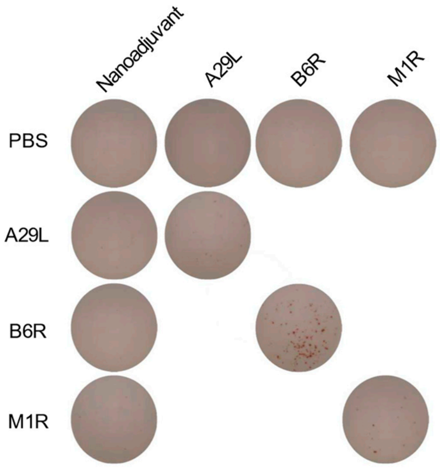

Figure S1. **Representative IFN-γ ELISpot images from splenocytes collected on day 14 post-boost.** Splenocytes were restimulated ex vivo with the corresponding recombinant antigen (A29L, B6R, M1R) or PBS control. Antigen-specific responses were visualized as spot-forming cells (SFCs), corresponding to the quantitative data shown in the accompanying analysis (n=3 per group).

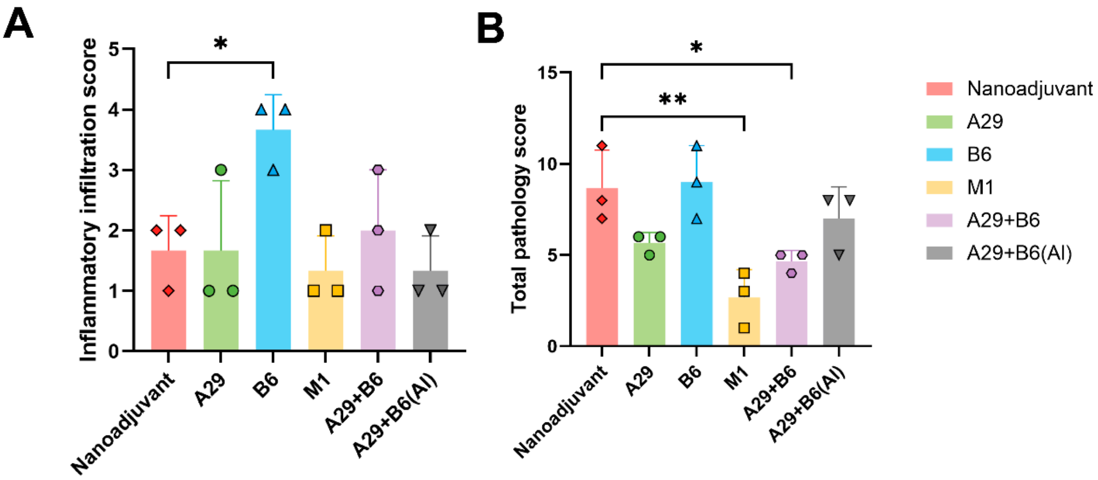

Figure S2. Lung histopathology scoring following MPXV challenge. (A) Inflammatory infiltration scores in lung tissues collected at the indicated time 5 dpi. Individual data points represent scores from individual mice (n = 3), with bars indicating mean ± SD. (B) Total pathology scores, including inflammatory infiltration, alveolar damage, epithelial degeneration, and hemorrhage. Statistical significance was determined using one-way ANOVA with appropriate post-hoc tests. *$p < 0.05$; **$p < 0.01$.
